# Mechanical and Thermal Properties of Hybrid Fibre-Reinforced Concrete Exposed to Recurrent High Temperature and Aviation Oil

**DOI:** 10.3390/ma14112725

**Published:** 2021-05-21

**Authors:** Muhammad Monowar Hossain, Safat Al-Deen, Md Kamrul Hassan, Sukanta Kumer Shill, Md Abdul Kader, Wayne Hutchison

**Affiliations:** 1 School of Engineering and Information Technology, University of New South Wales, Canberra 2610, Australia; muhammad.m.hossain@student.adfa.edu.au (M.M.H.); mdkamrul.hassan@student.adfa.edu.au (M.K.H.); 2 Civil Engineering Department, Military Institute of Science and Technology, Dhaka 1216, Bangladesh; 3 School of Engineering, Deakin University, Geelong 3220, Australia; sukanta.shill@deakin.edu.au; 4 Department of Applied Mathematics, Research School of Physics, Australian National University, Canberra 2600, Australia; mdabdul.kader@anu.edu.au; 5 School of Physical, Environmental and Mathematical Sciences, UNSW, Canberra 2610, Australia; w.hutchison@adfa.edu.au

**Keywords:** hybrid fibre-reinforced concrete, thermal conductivity, spalling, residual mechanical strength

## Abstract

Over the years, leaked fluids from aircraft have caused severe deterioration of airfield pavement. The combined effect of hot exhaust from the auxiliary power unit of military aircraft and spilt aviation oils have caused rapid pavement spalling. If the disintegrated concreted pieces caused by spalling are sucked into the jet engine, they may cause catastrophic damage to the aircraft engine or physical injury to maintenance crews. This study investigates the effectiveness of incorporating hybrid fibres into ordinary concrete to improve the residual mechanical and thermal properties to prevent spalling damage of pavement. Three fibre-reinforced concrete samples were made with micro steel fibre and polyvinyl alcohol fibre with a fibre content of zero, 0.3%, 0.5% and 0.7% by volume fraction. These samples were exposed to recurring high temperatures and aviation oils. Tests were conducted to measure the effects of repeated exposure on the concrete’s mechanical, thermal and chemical characteristics. The results showed that polyvinyl alcohol fibre-, steel fibre- and hybrid fibre-reinforced concrete suffered a 52%, 40% and 26.23% of loss of initial the compressive strength after 60 cycles of exposure to the conditions. Moreover, due to the hybridisation of concrete, flexural strength and thermal conductivity was increased by 47% and 22%. Thus, hybrid fibre-reinforced concrete performed better in retaining higher residual properties and exhibited no spalling of concrete.

## 1. Introduction

Concrete is a versatile building material of the 21st century. It is a durable material with excellent mechanical and thermal properties and has broad structural application. However, it has a significant drawback, being a quasi-brittle material, and an increase in the strength also increase its brittleness [1]. Moreover, ordinary concrete possesses lower tensile strength and propagates cracks easily when subjected to severe loading conditions [1,2,3]. Modern construction works demand concrete with a combination of qualities such as higher strength, excellent thermal properties, higher durability, and toughness [1,4,5]. The recent improvement in modern concrete, including high strength concrete (HSC), and fibre-reinforced concrete (FRC), offer some outstanding properties [4,5].

The production of HSC concrete requires the use of supplementary cementitious materials, such as ground granulated blast furnace slag, silica fume and fly ash as a partial replacement of ordinary Portland cement (OPC) [6,7]. The addition of pozzolans in concrete decreases the porosity. The extra water inside the concrete pore turns into vapour when exposed to a high temperature. This extra vapour pressure increases the risk of spalling in concrete [8] due to the lack of required porosity. Concrete is also susceptible to crack development due to several reasons, such as plastic shrinkage, drying shrinkage, corrosion cracks, and thermal cracks, and these cracks continue widening over the lifetime of concrete [9,10]. Subsequently, the concrete microstructure is exposed to harmful substances such as moisture, sulphates, chloride and different oils [4,11].

Earlier studies [12,13,14] have reported that HSC is susceptible to explosive spalling damage due to high temperature. Spalling is defined as a sudden break down of concrete into small pieces/flakes due to high-temperature exposures. The underlying reasons for the spalling effect are not yet fully comprehended. Few studies [13,14,15] suggested that internal cracking may be the primary cause of spalling. The fire triggers high pore pressure, dehydration and cement decomposition, which develops the cracks in the cement paste and aggregates [16,17]. Moreover, rapid heating may cause a heat disparity between the surfaces (exterior and interior) of concrete, which possibly initiate the internal cracks. Besides, some researchers stated that cracks originate from the pore inside the concrete [18,19]. Other significant factors affecting the concrete’s spalling damage are moisture content and strength grade.

Researchers in the last few decades saw a sharp rise in the use of different fibres in concrete to increase its mechanical properties such as flexural strength, toughness, tensile strength and durability [20,21,22,23,24]. The fibres’ effectiveness usually depends on fibre, fibres geometry and orientation, volume fraction (V_f_) and properties of fibres matrix in the concrete mixture [25,26]. Among the commercially available fibres, steel, carbon and polymer fibres are the most widely used ones [27]. Although the addition of one fibre in normal concrete enhances the properties of concrete to a certain level, the addition of multiple fibres significantly increases the properties of concrete. Thus, a careful design of the composite materials is essential to ensure a beneficial interaction between the fibres [28,29,30].

**Figure 1:**
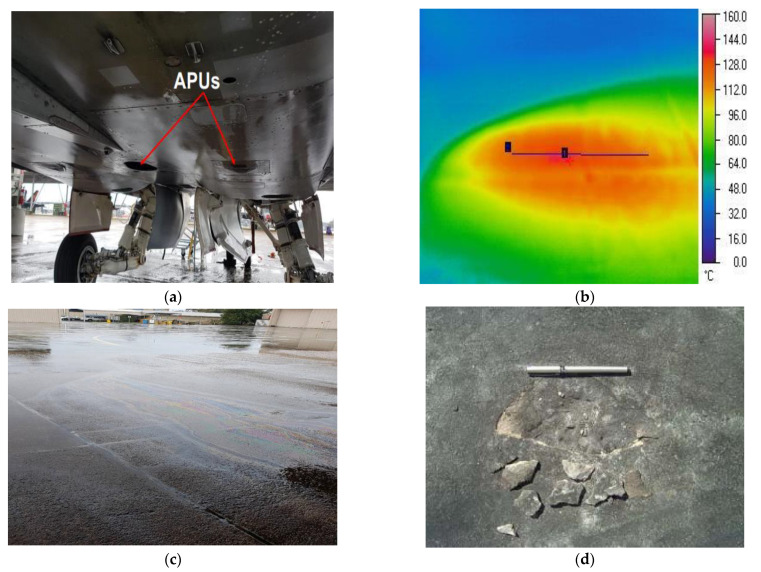
(**a**) APU of a F/A-18 Hornet aircraft, (**b**) thermal profile of military airfield pavement exposed to APU exhaust, (**c**) oil leak after single maintenance, and (**d**) spalling of the parking apron.

Most aprons and runways of military airbases are constructed using OPC concrete. These aprons and runways face more severe operating conditions than ordinary commercial airports because of the powerful modern aircraft such as the Harrier jet, F/A-18 and B-1. These pavements are repeatedly subjected to hot exhaust from an auxiliary power unit (APU) of modern aircraft, and aviation oils spill either in conjunction or separately, as seen in Figure 1. For smooth engine functioning, the aircraft use aviation oils, primarily hydrocarbons (HC) compounds, such as jet oils, hydraulic oils and engine lubricants. While carrying out routine maintenance or refuelling, leakage from civilian/military aircraft cause spilling of aviation oils on rigid pavements [31,32]. Worldwide, the rigid pavement under the Harrier jet, B-1 and F/A-18 aircraft have been experiencing pavement damage issues since the induction of these aircraft in the early days of 1980 [31,32,33]. Lee et al. [32] recorded that the APU’s exhaust, which provides power to the main engine of F/A-18, impinges the pavement at 45° angles and raises the pavement temperature to 175 °C, as seen in Figure 1a,b. The average exposure to APU exhaust is 15 min. The spalling of concrete pavement exposed to APU of a F/A-18 Hornet aircraft was also reported by Lee et al. as seen in Figure 1d. The combined effect of APU exhaust impinging on the reinforced concrete surface and spilled HC fluids causes the concrete strength’s deterioration, resulting in spalling of the top layer.

A few earlier studies also reported the causes of the spalling damage of airfield pavements exposed to repeated high temperature and HC fluids. However, these studies focused on causes like vapour pressure, residual mechanical and strength degradation for the chemical reactions. Currently, there are no research data on spalling damage, residual mechanical and thermal properties of FRC under similar conditions. Such data play a significant role in selecting fibre type, volume fraction, fibre matrix and geometry while designing for airfield pavements. To fill this knowledge gap, this study investigates the effectiveness of incorporating hybrid fibres into ordinary concrete to improve the residual mechanical and thermal properties to prevent spalling damage. In this investigation, the different combination of micro steel fibre (SF), polyvinyl alcohol fibre (PF) and hybridisation of PF and SF are produced to examine the effect of two fibres in FRC in comparison with ordinary concrete. Moreover, to study the effect of hybridisation of concrete using PF and SF, three mixed fibre-reinforced concrete are produced with a fibre content of 0.3%, 0.5% and 0.7% from each type. All fibre-reinforced samples are compared with the control specimen that contains no fibre. The residual compressive strength, flexural strength, mass loss and spalling effect after repeated exposure to high temperature and HC fluids are evaluated. This study also explored the thermal performance of FRC and its effect on spalling due to repeated exposure to HC fluids and high temperature. 

## 2. Materials and Methods

### 2.1. Materials and Mixing Procedure

**Figure 2:**
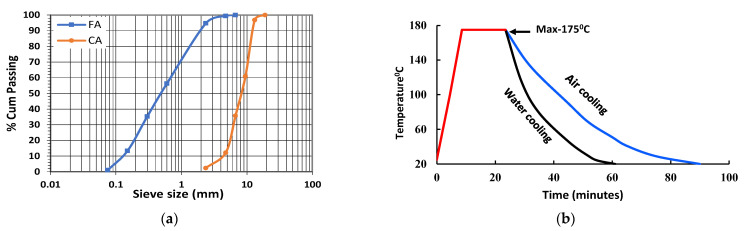
(**a**) Gradation curves of aggregates, (**b**) heating and cooling cycles of specimens.

**Figure 3:**
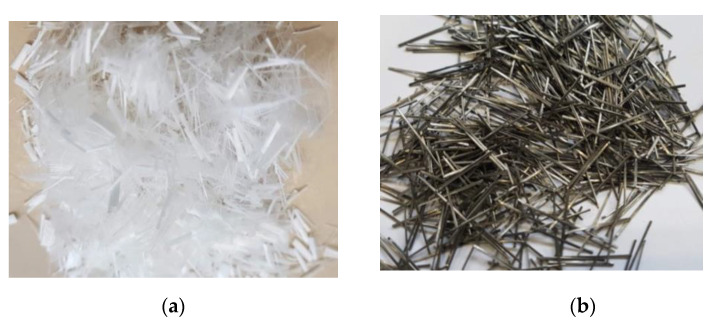
Types of fibres used; (**a**) PF and (**b**) SF.

Fresh M70 grade concrete samples were prepared using Boral general-purpose (AS 3972) OPC with a water-cement (*w*/*c*) ratio of 0.42. Table 1 and Table 2 respectively describe the physical and basic properties of the OPC at ambient temperature. Maximum 10 mm-sized basalt coarse aggregate (CA) and local river sand (FA) with fineness modulus of 2.6 were used. The water absorption and specific gravity of the CA and FA were 0.34% and 2.66, and 0.72% and 2.62, respectively. Figure 2a shows the grading curves of the aggregates used in this experiment. ADVA 650, a synthetic carboxylated polymer, was used as a superplasticiser with variable dosages to maintain the slump around 120 mm. Straight-end 8-mm long SF and 12-mm long PF fibres were used. Figure 3 and Table 3 respectively show the geometry and properties of used fibres in this experiment. Cylinder specimens of 100 mm × 200 mm were cast to determine mass loss and spalling tests. Furthermore, 100 mm × 100 mm × 350 mm size beams were cast for the flexural test. Cube specimens of 50 mm × 50 mm × 50 mm were used to measure the residual compressive strength. When samples are exposed to HC fluids, it penetrates the concrete from all-direction, leaving a thin core intact in the cube samples, thus depicting the critical condition corresponding to residual compressive strength. Samples curing continued for four weeks in a fog room with relative humidity (RH) > 90% and 23 ± 1 °C. After 28 days of curing, all mechanical and thermal properties were tested. Then some samples were exposed to repeated high temperature only, and other samples were exposed to the combined effect of high temperature and HC fluids. The thermal properties of specimens were measured each after 20 cycles of exposures. In all tests, an average of the data of three samples was recorded. The mix design for different FRC specimens is given in Table 4. This table also indicates the percentage of superplasticiser used to mix the cementitious materials’ total weight.

### 2.2. Exposure to Recurring High Temperature and HC Fluids

After 28 days of curing, all samples were exposed to two recurring thermal conditions: High-temperature cycles without oil exposure and combined actions of HC fluids and high temperature. According to McVay et al. [33], airfields are usually exposed to different aviation oils such as engine oil, hydraulic oil and aviation fuel. In this experiment, AeroShell Turbine Oil 500, AeroShell Fluid 31 and jet fuel (F-34 kerosene grade) were mixed in equal parts and sprayed on the specimens before each exposure to high temperatures. HC fluid-soaked samples were kept in a high-temperature electric oven for 15 min at 175 °C. The heating rate, duration of a thermal cycle and cooling procedures are presented in Figure 2b. Along with the inbuilt oven thermocouple, an external thermocouple was also used to monitor the sample surface temperature. After 15 min of heat exposure, the hot samples were cooled down by air and, once in a week, were cooled down by spraying water to simulate rainfall effects on airfield pavements. Recurrence of oil spray, heating and the cooling cycle continued until spalling occurred. Three samples of each type were used to determine different mechanical and thermal properties after every 20 cycles of exposure. The notation E0, E20, E40 and E60 denote the number of exposure cycles mentioned in individual cases.

### 2.3. Experimental Procedures

#### 2.3.1. Mechanical Properties

The samples’ initial and residual compressive strength was measured as per AS 1012.9–1999 [34]. Samples weights and densities were measured in saturated surface dry condition. Then they were placed in the furnace at 105 °C until they reached a constant dry weight. A universal hydraulic testing machine with a loading rate of 0.3 MPa/s was used for the compressive strength test. 

#### 2.3.2. Four-Point Bending Test

**Figure 4:**
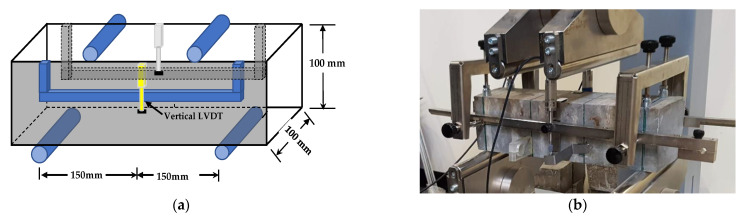
(**a**) The flexural strength test setup, (**b**) FRC specimen during the test.

Beam samples were assessed for flexural strength, according to AS 1012.11. Control’s (manufacturer) flexural testing machine of 150 KN capacity with a deflection rate of 0.3 mm/min was used to measure the concrete beams’ flexural strength. Figure 4a,b represents the test setup and the actual specimen test. Two transducers were attached with brackets to the beam specimen to measure the mid-span deflection, taken as the two readings’ average. Two metallic cylinders positioned at 100 mm from the supports (1/3 distance of the span) were used to apply the load equally. Two identical metallic cylinders spaced at 300 mm were used to support the specimen.

#### 2.3.3. Measurement of Thermal Properties

The specimens were assessed for thermal properties like specific heat and thermal conductivity each after 20 exposures. As per ASTM C518 [35], the Netzsch (manufacturer, Selbu, Germany) Heat Flow Machine (HFM) 446 Lambda machine was used to test 150 mm × 150 mm × 25 mm samples for thermal conductivity and specific heat. A temperature gradient difference of 20 °C between two plates was used while measuring the thermal properties. Two heat-flow sensors fixed in the plates were used to measure the heat flow into the material and out of the material, respectively. 

#### 2.3.4. X-ray Diffraction (XRD) Analysis

The XRD test was used as a non-destructive method to determine crystal phases and mineral compounds present in the specimens [36]. The samples’ crystalline phases were identified between 10 to 70 (2θ), applying a scanning speed of 0.2 degrees/min. The selected specimen was crushed using a ball mills machine, then sieved through a screen aperture of 75 μm. HC fluid exposed samples were oven-dried for 24 h before grinding them into powder. XRD instrument Rigaku Miniflex 600, operated at 40 kV and 45 mA, was used to record the crystal patterns using CuKα (λ = 0.15400 nm) radiation [37,38].

#### 2.3.5. Thermogravimetric (TG) Analysis

After 20 cycles of high-temperature exposures, the differential scanning calorimetry (DSC) and TG tests for the specimens were conducted using NETZSCH (manufacturer) STA 449C Jupiter, Selbu, Germany. The DSC and TG spectra were collected in an inert nitrogen environment with a heating rate of 10 °C/min from 20 to 800 °C. 

#### 2.3.6. Microstructure Investigation

The Zeiss Axio Imager (Zeiss, Oberkochen, Germany) optical microscope was used to analyse fibre reinforced samples’ microstructures at the original condition and after 60 cycles of high-temperature and HC fluid exposures. This machine analyses the microcrack and voids development in the samples due to high temperature and HC fluids’ simultaneous effect. For microstructural analysis, samples were collected from the top (up to 20 mm) surface of the specimens. The samples’ microstructure is also analysed by combining two main techniques: Direct observation with an optical microscope and scanning electron microscope (SEM). This combination method allows a better understanding of links between microstructure, composition and engineering behaviour.

## 3. Results and Discussion

### 3.1. Effect of HC Fluids and Repeated Thermal Exposure on the Residual Mechanical Properties

#### 3.1.1. Residual Compressive Strength

**Figure 5:**
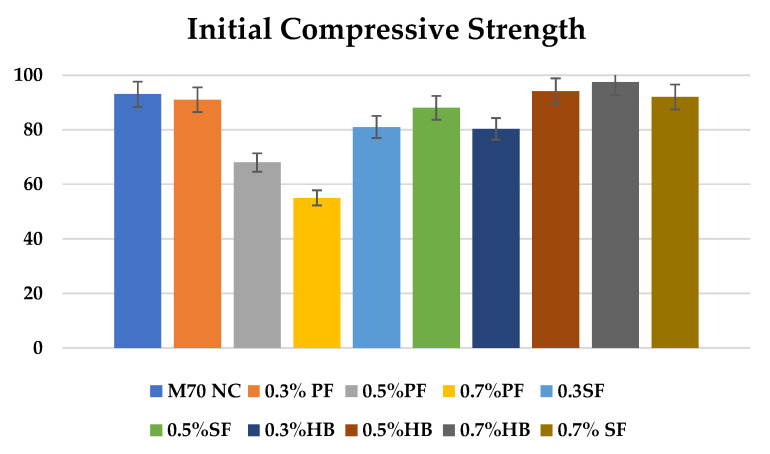
Initial compressive strength of fibre-reinforced concrete.

**Figure 6:**
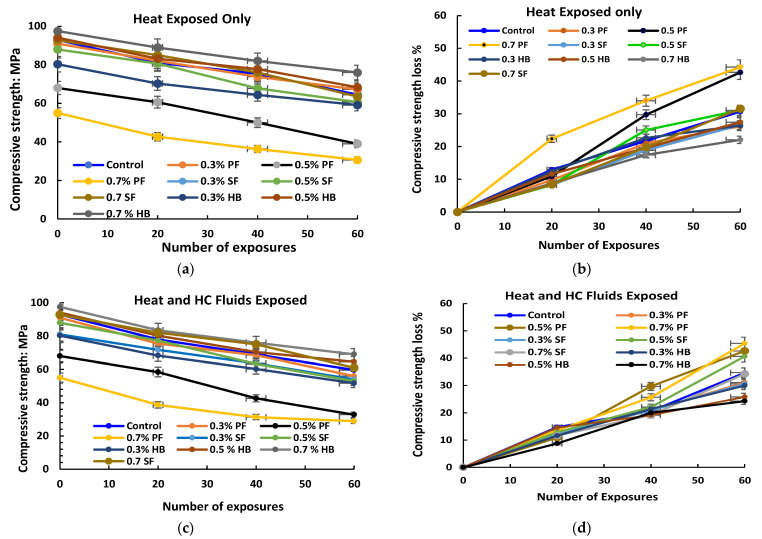
Residual compressive strength. (**a**) Heat exposed only samples, (**b**) % loss of compressive strength of heat exposed samples only, (**c**) heat and HC fluid exposed samples, (**d**) % loss of residual compressive strength of heat and HC exposed samples.

Various factors govern the behaviour of samples exposed to higher temperature and HC fluids. Figure 5 shows the initial compressive strength for different fibre-reinforced concrete. Figure 6 shows the residual compressive strength and % loss of compressive strength of samples exposed to high temperature only and the combined effect of high temperature and HC fluids. For repeated exposure up to 175 °C only, loss of significant compressive strength was recorded, as seen in Figure 6a,b. After subjecting specimens to a recurring high temperature for 60 cycles, the control specimens lost 32.65% of their initial compressive strength. For 0.3% SF and 0.5% SF reinforced specimens, compressive strength loss was 26.50% and 31.06%, respectively. For PF reinforced concrete, the loss was more significant. For 0.3%, 0.5% and 0.7% PF reinforced concrete, the compressive strength loss was 26.15%, 42.65% and 44.24%, respectively. The addition of extra PF would create more porosity inside the concrete matrix after melting, resulting in more deterioration of its residual properties. Whereas for SF, no melting of SF occurs at 175 °C, thus no extra porosity is created that reduces concrete strength significantly. Similarly, for HB specimens with 0.3%, 0.5% and 0.7% HB fibre reinforcement, the compressive strength loss was 27.42%, 25.40% and 22.05%, respectively.

**Figure 7:**
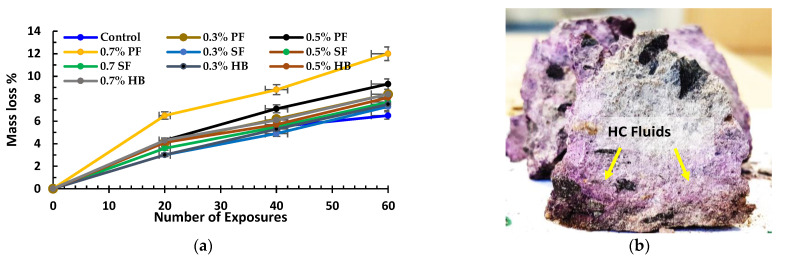
(**a**) Mass loss of specimens after 60 cycles of high temperature and HC fluids, (**b**) penetration of HC fluids inside the 0.3% PF cube specimens after E60.

For repeated exposure to the high temperature and HC fluids’ combined effect, residual compressive strength loss was more than only heat-exposed samples, as seen in Figure 6c,d. Penetration of aviation oil after 60 cycles is identified as pink and dark colours in the crashed cube specimen, as shown in Figure 7b. After 60 cycles of combined exposures, the control specimens lost 35.91% of their initial compressive strength. For 0.3% and 0.5% SF reinforced specimens, compressive strength loss was 33.33% and 39.77%, respectively. For PF reinforced concrete, the loss was more significant. For 0.3%, 0.5% and 0.7% PF specimens, the compressive strength loss was 38.46%, 47.45% and 51.84%, respectively. After repeated exposure to high temperature and HC fluids, heat caused PF fibres to melt. Those melted channels allow more HC fluids to penetrate the concrete, which causes significant strength reduction. Similarly, for HB specimens with 0.3%, 0.5% and 0.7% HB fibre reinforcement, the compressive strength loss was 35.55%, 31.46% and 26.23%, respectively. In general, high-temperature and HC fluids exposed samples suffer more compressive strength loss due to chemical reaction between the cement and aviation oils to form harmful salts. S.k. Shill et al. [39] also reported similar strength loss due to the chemical reaction between cement and ester, the fatty acid of HC fluids. However, under similar circumstances, HB fibre reinforcement concrete suffered comparatively lower strength loss.

In summary, PF fibres in HB concrete created additional microchannels allowing the release of vapour pressure created by exposure to HC fluids. The inclusion of SF also increases the tensile capacity [38]; thus, this matrix helped to retain more residual compressive strength than the control specimens. 

#### 3.1.2. Mass Loss

The mass loss of specimens after recurring exposure to high temperature and HC fluids is shown in Figure 7a. The specimens lost their initial free water entirely due to recurrent high temperature within the first few cycles. Within the first twenty cycles of heating, the mass loss rate was higher due to the evaporation of initial free water, which escaped outside in a vapour state. After 20 cycles of HC fluids and high-temperature exposures, the mass loss slope decreases gradually, which implies that the concrete matrix lost its initial free water within the initial cycles. However, the maximum fibre V_f_ = 0.7% used in this study was low enough to cause any significant mass loss due to fibres’ presence. Specimens containing 0.7% PF fibres and 0.7% SF suffered a mass loss of 12% and 7.7%, respectively. At the same time, HB samples lost 8.4% of the mass after 60 cycles. However, Lee et al. [32] suggested that below 200 °C, loss of cementitious component is negligible. Nevertheless, a few other studies [40,41] also strongly support that when concrete is subjected to chemical and high temperature, the mass loss could be due to the decomposition of mineral compounds in concrete besides the loss of initial water.

In summary, initial mass loss occurs due to the evaporation of initial free water for exposures to high temperature and HC fluids. Subsequent mass loss occurs due to the evaporation of chemically bonded water, decomposition of C-S-H gel and calcium hydroxide, which is also identified in the XRD and TG analysis paragraphs of this paper.

#### 3.1.3. Flexural Strength

**Figure 8:**
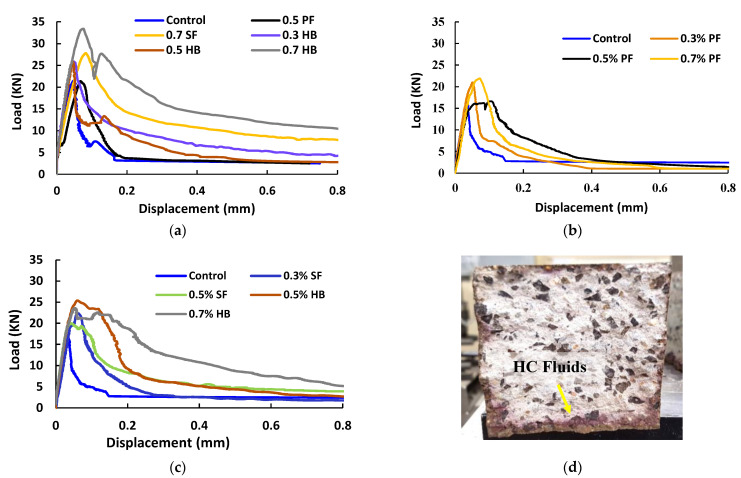
Typical load vs. displacement curve (combined exposures). (**a**) Samples after E60, (**b**) PF samples after E60, (**c**) SF and HB FRC after E60, (**d**) HC fluid penetration into beam after E60.

Control and FRC samples were evaluated for flexural strength after E0 and E60 cycles of high temperature only and combined high temperature and HC fluid exposures. The average of three sets of data was used to calculate the flexural strength. The midspan deflection against the applied load under four-point bending is shown in Figure 8 for control, PF, SF and HB specimens. The flexural strength of the control specimen at E0 was 5.08 MPa. The inclusion of fibres in the concrete matrix increased the flexural strength and increases in the V_f_ further improved the flexural load capacity of concrete. For samples without any heat and HC fluids exposures (E0), the inclusion of 0.3%, 0.5% and 0.7% PF fibre led to the flexural strength increasing by 4.5%, 15.89% and 21.82%, respectively. With the inclusion of 0.3%, 0.5% and 0.7% SF fibre, the flexural strength increased by 15.5%, 17.89% and 22.82%, respectively. Similarly, with the inclusion of 0.3%, 0.5% and 0.7% HB fibre, the flexural strength increased by 13.13%, 17.89% and 32.29%, respectively. The flexural strength and descending branch after peak load were significantly improved in concrete reinforced with SF and HB compared to PF fibre.

The flexural capacity of samples exposed to a high temperature and HC fluids decreased significantly, as shown in Figure 8b,c. The penetration of HC fluids into the beam sample is shown in Figure 8d. As discussed earlier, due to the melting of PF fibre and deterioration of tensile strength of fibres due to high temperature and HC fluid exposure, significant flexural strength loss was recorded. After 60 cycles of high-temperature and HC fluids exposure, the control specimen lost 23% of the initial flexural strength. For 0.3%, 0.5% and 0.7% PF specimens, flexural strength loss was 6.11%, 13.13% and 21.23% of their initial strength, respectively. For 0.3%, 0.5% and 0.7% SF specimens, flexural strength loss was 9.67%, 7.84% and 6.91%, respectively, and for 0.3%, 0.5% and 0.7% HB specimens, flexural strength loss was 18.38%, 16.93% and 11.59%, respectively.

In summary, the residual flexural strength and the post-peak deformation behaviour of FRC were significantly improved with PF and SF. Due to the hybridisations, high-temperature and HC fluids exposed samples’ maximum residual flexural strength loss was 23% for control and 11.59% for 0.7% HB sample.

#### 3.1.4. Visual Observations of Ruptured Sections of FRC

**Figure 9:**
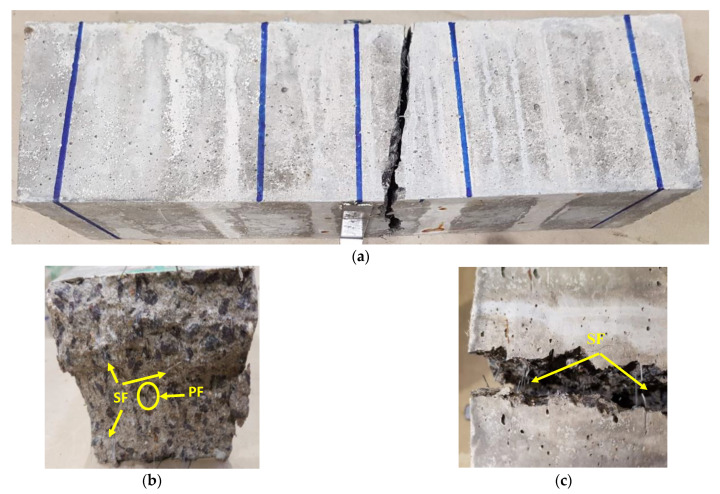
Rupture sections after E0. (**a**) Crack pattern of 0.5% SF beam after four-point loading test, (**b**) ruptured cross-section of 0.7% HB FRC sample, (**c**) ruptured cross-section of 0.5% SF sample.

The ruptured sections of the FRC were carefully inspected after the flexural test. The increase in fibre fraction resulted in the smoothness of ruptured sections for FRCs but was reduced with an increase in high temperature and HC fluids exposures. The fracture energy between the cement pastes and aggregates interface decreased with high temperature and HC fluid exposures because high temperature decreases the bond between the cement paste and aggregates. Figure 9 shows the typical ruptured cross-section of the hybrid FRC specimen after the flexural test at E0. However, regarding the fracturing process concerned, there are no significant differences between the fractured faces. Thus, it suggests that fibre content or types do not play a significant role in fracture mode. FRC specimens exposed to 60 cycles of high temperature and HC fluids also failed with the same fracture modes, which means that temperature has no significant effect on fracture modes.

However, PF FRC samples were split entirely into two parts since fibres were melted after repeated heating as also reported in SEM results, thus behaving like ordinary concrete. The SF has been seen protruding from the concrete matrix after the specimen has ruptured, as seen in Figure 9b,c; the PF, once it reached its maximum tensile strength, also ruptured. Pliya et al. [42] and Çavdar et al. [43] also found that SF and HB fibres provide more flexural strength than single PF fibre-reinforced concrete.

#### 3.1.5. Concrete Spalling

**Figure 10:**
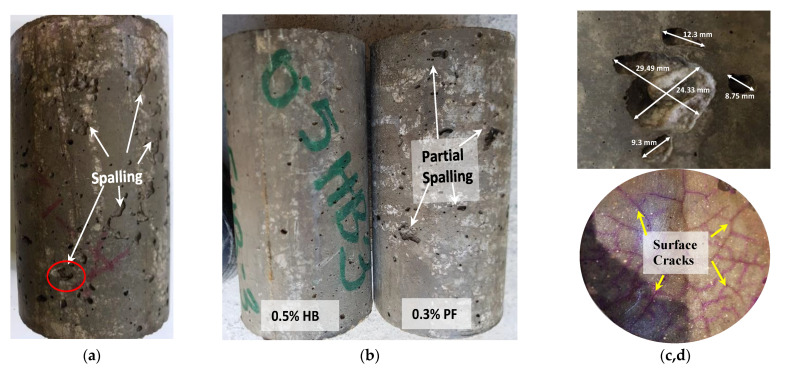
Spalling of specimens after 60 cycles of high temperature and HC fluids exposures; (**a**) control specimen, (**b**) no significant spalling in HB sample, and partial spalling in PF sample, (**c**) enlargement of a spalled section of control specimen, (**d**) surface cracking of spalled samples.

Spalling of concrete has a significant influence on concrete fire performance and can be a governing factor in determining the fire resistance of an RC structural member [43]. Some researchers [18] reported spalling in high-strength concrete once subjected to high temperatures. Similarly, ordinary concrete specimens exposed to recurring 60 cycles of high temperature and HC fluids suffered significant spalling, as seen in Figure 10. Though samples were exposed to 175 °C, the combined effect of chemical degradation due to aviation oils and repeated heating may have caused the spalling. PF samples also suffered partial spalling (Figure 10b) once exposed to high temperatures and HC fluids. However, no significant spalling in HB FRC samples. The main reason for spalling in the high strength concrete is its dense microstructure, which prevents moisture from escaping when exposed to high temperatures. In this study, the lack of spalling phenomenon in the hybrid fibre-reinforced samples’ might be due to the melting of PF fibres, thus creating a microchannel, which helps release vapour pressure, and the SF prevents the spalling by stopping micro-crack propagation.

### 3.2. Measurement of Thermal Properties

Concrete is an anisotropic and non-homogenous material where hydrated cement paste helps to keep aggregates held together. Due to its porosity, moisture content affects the pore conductivity of concrete. Thermal properties of concrete have a significant role in retaining its strength because the concrete structure deteriorates quickly once exposed to high temperature. In this study, the thermal conductivity and specific heat of FRC and control specimens subjected to recurrent higher temperatures and HC fluids were measured.

The experiment involves measuring the amount of heat flow q through the sample once the equilibrium condition is achieved. Thermal conductivity is k, the sample thickness is Δx, the temperature difference across the sample is ΔT and cross-sectional area is A through which heat flows influences the magnitude of the heat flow q. Fourier’s law of conduction gives the relation between these parameters:

Q = K·A·(ΔT/Δx)(1)

**Figure 11:**
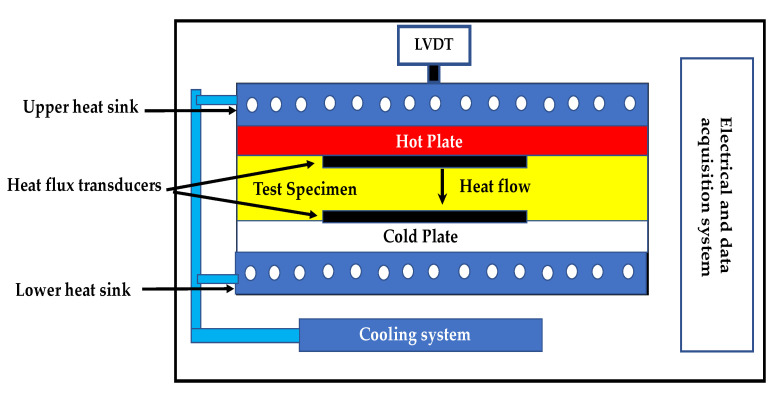
Measuring principle of HFM 436.

Two heat flow transducers of the HFM 436, the heat flow instrument used to calculate the amount of heat flow (in volts) through the sample, is shown in Figure 11. The area through which heat flows is the same as the area measured by the heat flow transducer and remains the same for all samples. Therefore:

q = NV(2)

N is the calibration factor related to the heat flow transducer’s voltage to the heat flux through the sample. Solving Equations (1) and (2) for k, the equation of thermal conductivity may be expressed in-unit W/m.k:

K = (N·V/A)·(ΔT/Δx)(3)

For calculation of thermal conductivity and specific heat, ASTM C518 and IS EN 12667/12939 [44] was followed. 

**Figure 12:**
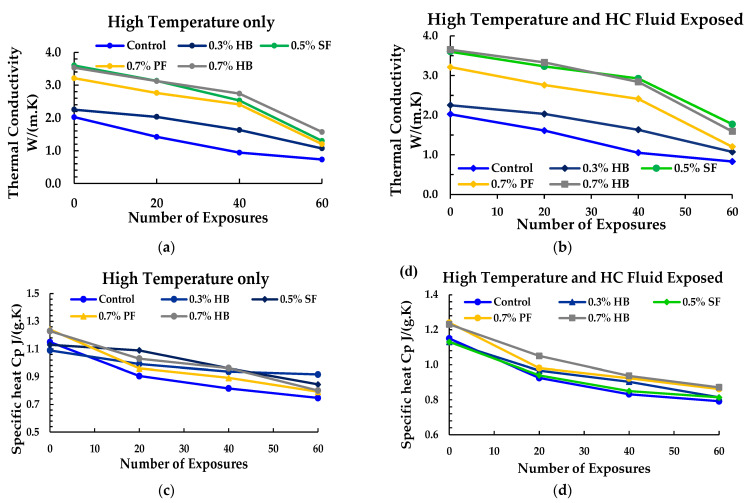
Thermal conductivity: (**a**) Only heated samples, (**b**) high temperature and HC fluids exposed; specific heat: (**c**) Only heated samples, (**d**) high temperature and HC fluid exposed samples.

Thermal conductivity measured for specimens exposed to high temperature only, and high temperature and HC fluids is shown in Figure 12a,b. Thermal conductivity of PF, SF, and HB FRC ranges between 1.41 to 3.53 W/m·k compared to 2.02 W/m·k of the control specimen. HB and SF samples showed higher thermal conductivity due to the presence of micro steel fibre, which is an excellent conductor of heat [39]. With the increase in the number of heat exposures, a significant decrease in thermal conductivity was recorded. This thermal conductivity trend may be due to the rapid decrease in moisture level following the evaporation of pore and free water of cement paste due to recurrent high-temperature exposures [45]. A similar thermal conductivity reduction due to high-temperature exposure was also reported by Kodur et al. [46]. However, samples exposed to repeated high temperature and HC fluids showed higher thermal conductivity than samples exposed to heat only. This tendency of slightly higher thermal conductivity may be attributed to the repeated exposure to HC fluids, which causes an increase in the samples’ moisture content.

A similar separate test was done to measure the value of specific heat Cp. The specific heat measured for specimens exposed to high temperature only and high temperature and HC fluids is plotted in Figure 12c,d against the number of exposures. The specific heat of PF, SF and HB FRC ranges from 1.03 to 1.4 compared to 1.15 of the control specimen. Kodur et al. [46] also reported lower specific heat values in ordinary concrete than the HSC. With the increase in the number of heat exposures, a significant decrease in the specific heat was recorded. SF and hybrid FRC show slightly higher specific heat values, which may be attributed to steel fibres. The use of PF fibres reduces the specific heat in comparison to other fibres.

Moreover, the deterioration of concrete due to repeated high temperature and HC fluids exposures results in hairline cracks, which increased PF porosity and other FRC porosity. In contrast, ordinary concrete has lower specific heat because of its lower permeability and dense microstructure, requiring additional heat to convert moisture into vapours.

### 3.3. XRD Analysis for FRC

**Figure 13:**
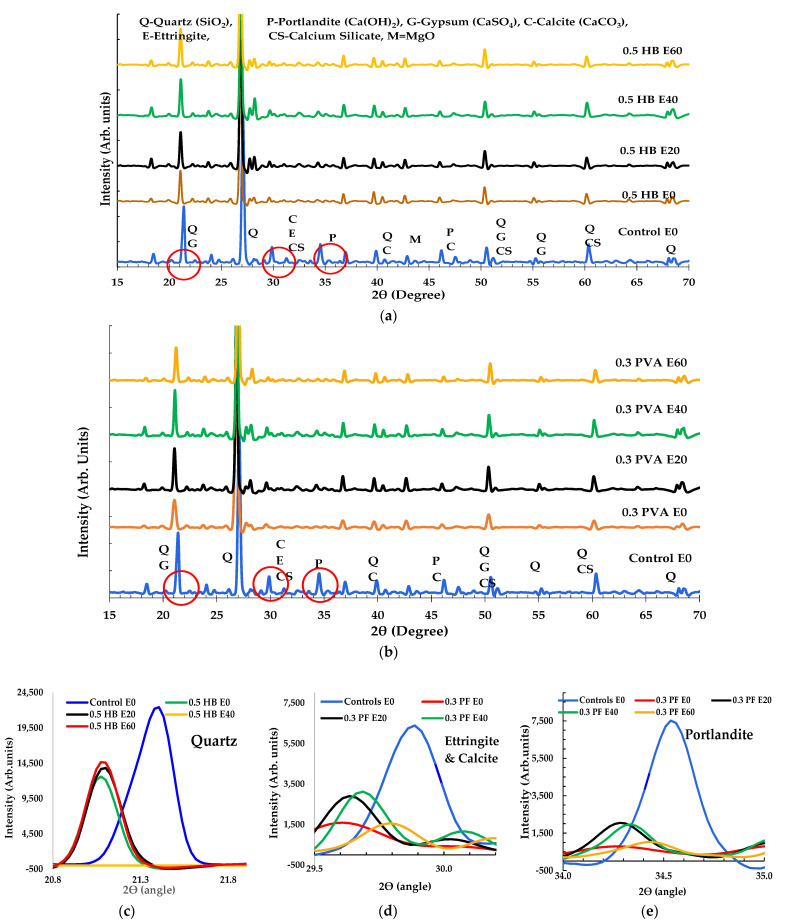
XRD of samples exposed to repeated heat and HC fluids (**a**) 0.5% HB FRC, (**b**) 0.3% PF FRC, zoom on the crystallographic modifications- (**c**) Quartz, (**d**) ettringite and calcium silicate and (**e**) portlandite.

The XRD peaks for control and FRC specimens appeared to be similar in original condition. This trend reveals that fibres do not contain any crystalline mineral compounds. Nevertheless, the intensity height of the ordinary concrete and FRC sample in XRD was found to be different in some cases. The addition of SF and PF in the concrete matrix might have caused the increase/decrease in the peak intensity height and peak shift for the crystalline compound in the XRD, as shown in Figure 13. The XRD results of the fresh ordinary concrete specimen show various crystal phases such as P = Portlandite, C = Calcite, E = Ettringite, Q = Quartz, G = Gypsum, M=magnesium oxide, alite C_3_S = 3CaO·SiO_2_, mullite = 3Al_2_O_3_2SiO_2_ and belite C_2_S = 2CaO·SiO_2_, similar results were also reported by other researchers [46,47]. According to Dittrich and Song et al. [18,48], when concrete is exposed to 80–90 °C due to thermal effect, ettringite in concrete can be decomposed and dehydrated.

Similarly, when exposed to 200 °C, physical and chemical decomposition in mineral compounds usually occurs. Recurrent exposure of control and FRC samples to 175 °C might cause the ettringite dehydration and decomposition. Similarly, the peak position of quartz, ettringite and portlandite have shifted to a lower degree (2θ), as shown in Figure 12c–e. Additionally, the minerals’ intensity height was significantly reduced. As stated by some researchers [44,45,46,49], this phenomenon may be due to the decomposition of crystal minerals when exposed to chemicals or high temperature. A few new peaks also appeared in the FRC specimens’ XRD pattern after repeated high temperature and HC fluids exposure. Shill et al. [50] reported that the chemical reaction of concrete with HC fluids may cause the formation of salt and soap compounds on the concrete surface, which may cause a few new peaks. 

However, a few of the picks like alite, mullite and calcite did not shift their position but a significant reduction in peak intensity was observed. Most mineral compounds in FRCs were affected by combined actions of HC fluid and a high thermal cycle.

### 3.4. Thermogravimetric (TG) Analysis

**Figure 14:**
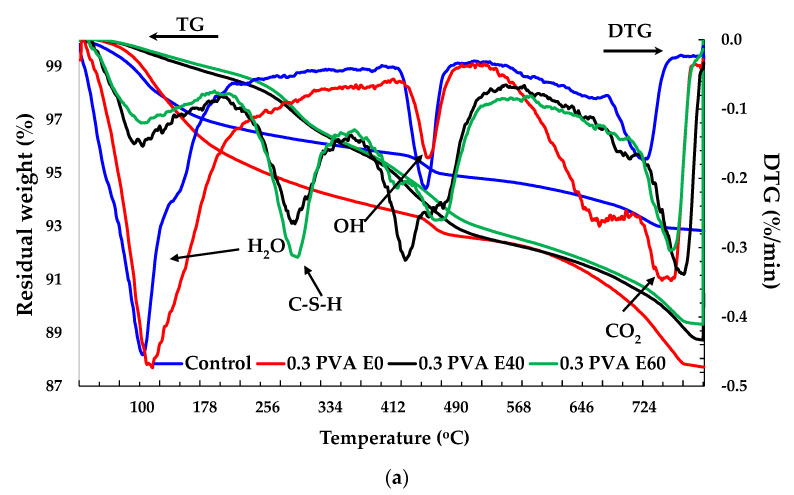

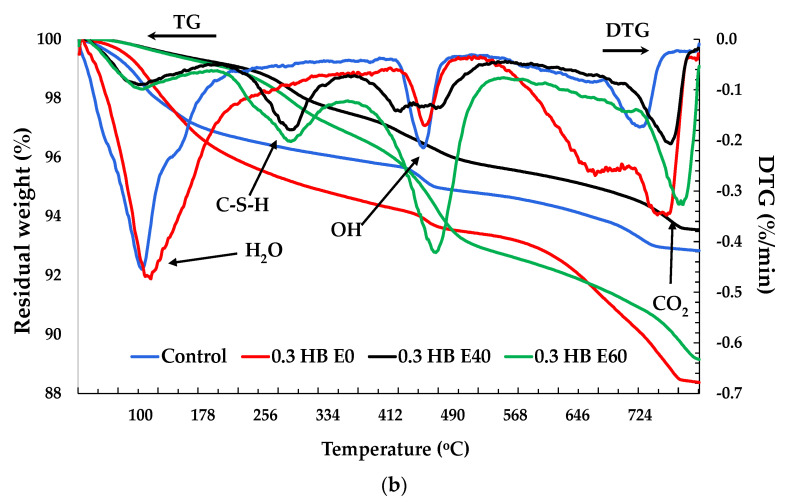
TG analysis of specimens after different cycles of exposures- (**a**) PVA specimens with 0.3% fibre content, (**b**) hybrid FRC specimens with 0.3% of MSF and 0.3% PVA fibre content.

The thermogravimetric (TG) tests were conducted for control and FRC specimens after HC fluid and high-temperature exposure, as shown in Figure 14. The TG curve of the original concrete showed three rapid weight loss sections. The picks between 20 to 200 °C represent weight losses due to the escape of parts of the bound water and free water, though Song et al and Fares et al. [51,52] suggested that the free water was eliminated at 120 °C. Then, at 182 °C, gypsum decomposed along with ettringite [45] and carboaluminate hydrates, causing mass loss. This sharp mass loss in the 30–200 °C temperature range was evaluated to be about 3.19%, 3.34%, 1.36% and 1.15%, respectively, for Control E0, 0.3% PF E0, E40 and E60. Repeated exposure to HC fluid and high-temperature cycles caused the reduction in mass loss because after initial exposure, excess and bound water almost dries up. Similarly, with the increase in the number of exposures, the mass loss percentage in higher temperature ranges increased significantly, as seen in Table 5.

The primary chemical reactions in different temperature range are given below:

(4) $${\mathrm{CaSO}}_{4}\cdot 2H_{2}O\;\to {\mathrm{CaSO}}_{4}\cdot \frac{1}{2}H_{2}O+\frac{3}{2}H_{2}O\cdot (120–150\;{}^{\circ}C)$$

Ca_6_[Al(OH)_6_]_2_(SO_4_)_3_·26H_2_O (Ettringite) → Ca_4_[Al(OH)_6_]_2_(SO_4_)·8H_2_O + 2CaSO_4_·0.5H_2_O + 17H_2_O (130–140 °C) (5)

The decomposition of the C–S–H and carboaluminate hydrates cause the loss of bound water from [52].

3.4CaO·2SiO_2_·3H_2_O (C–S–H) → 3.4CaO·2SiO_2_ + 3H_2_O·(180–300 °C) (6)

Decomposition of portlandite:

Ca(OH)_2_ → CaO + H_2_O (450–550 °C) (7)

Decarbonation of calcium carbonate [53]:

CaCO_3_ → CaO + CO_2_ (700–900 °C) (8)

After the initial rapid decline, the actual weight loss rate stabilised between 200 °C and 350 °C. The second significant weight loss was observed between 350–600 °C, where portlandite started decomposing until completion of the process at around 550 °C [54,55]. The mass loss in this range was recorded to be 5.54% in PF E60 and 3.49% in HB E60. However, after 400 °C, mass loss was significantly reduced in FRC than in control specimens. In the 650–800 °C range, the release of carbon dioxide in addition to the decomposition of C–S–H (II) into wollastonite and larnite, contributed to the mass loss, which accounts for 3.645%, 4.11% and 4.26% of mass loss in Control E60, PF E60 and HB E60, respectively.

### 3.5. DSC Analysis

**Figure 15:**
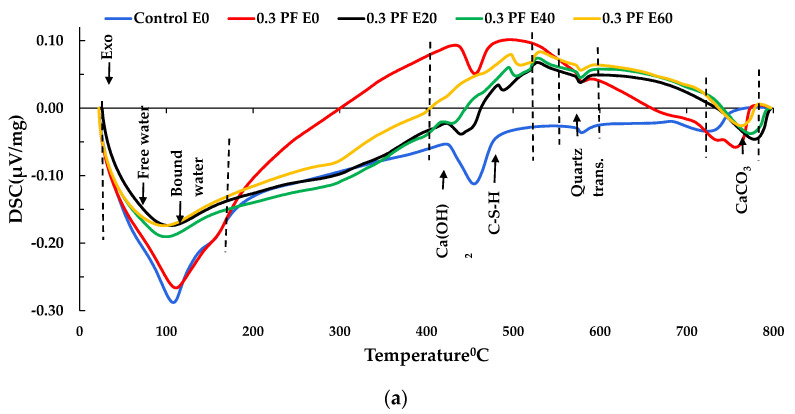

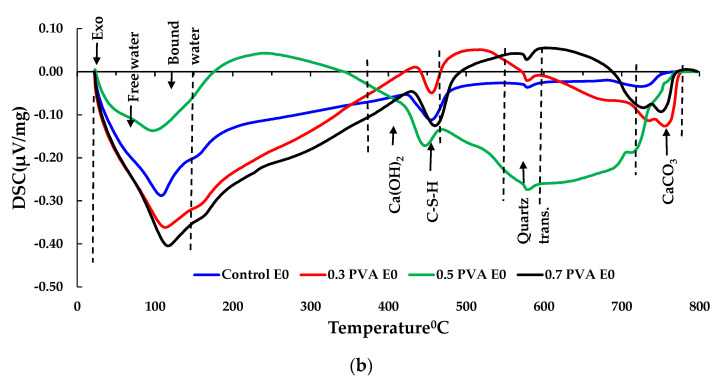
DSC test from 0 to 60 exposures (**a**) PF FRC, (**b**) HB FRC.

The DSC plots of the control, PF, and HB specimens are shown in Figure 15, supporting the TGA analysis’ claims. The DSC plot and TGA analysis of specimens show similarity in FRC decomposition and dehydration behaviour with other researchers [56,57]. The release of free water, bound water of C-S-H gel and ettringite was recorded up to 200 °C [55,58]. However, few differences in the heating process were observed between FRC and control samples. Notably, below 120 °C, both PF and HB FRC offered a lower peak than control. This phenomenon could be attributed to the lower unit weight of FRC compared to the control specimen that resulted in the increased volume of mixtures and, therefore, more free water in the specimens.

According to Noumowé and Li et al. [59,60], continuous dehydration of the C-S-H gel and melting of PF were seen between 200–300°C and at 480 °C, and a slight variation of heat flow was observed in those ranges. Between 400–500 °C, significant decomposition of Ca(OH)_2_ occurred with peak shifts with increased heat exposures. For recurrent exposure of FRC samples to high temperature, a significant reduction in peak intensity at 440 °C (in Figure 15a) was recorded in contrast to that of E0, particularly for 0.3% PF reinforced concrete after 60 exposures. This trend may be due to the continuous dehydroxylation of portlandite in the cement matrix. The phase transition α to the β phase of quartz was recorded at around 573 °C with a visible peak on DSC plots. Between 700–800 °C, decomposition of calcium carbonate occurs, which release carbon dioxide from the concrete. Sun and Xu [34] also found that between 700 and 800 °C, the calcium carbonate (CaCO_3_) decomposes, thus permitting CO_2_ to liberate from the concrete. 

### 3.6. Microstructure Analysis

**Figure 16:**
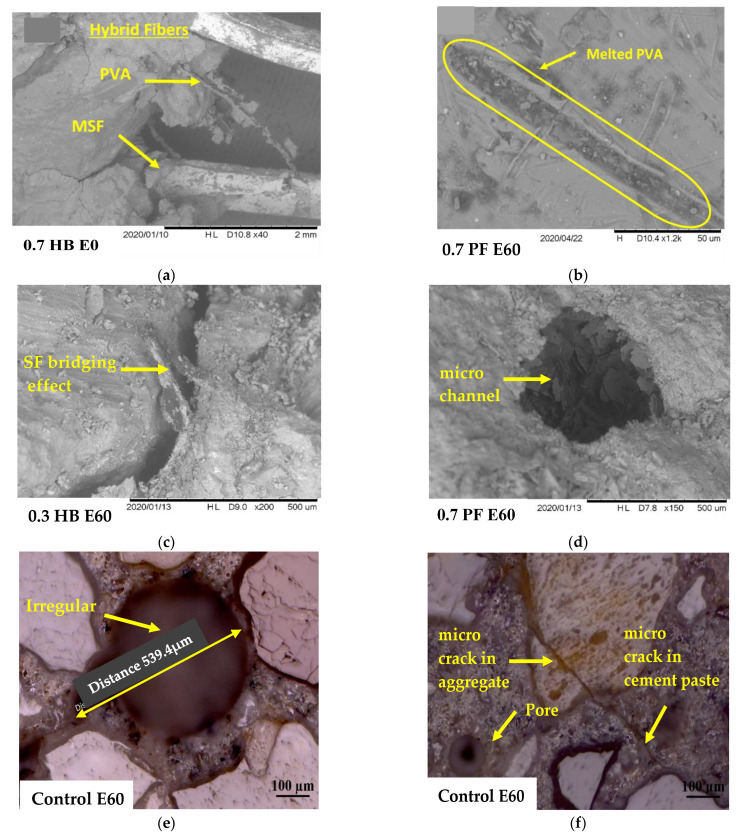
S.E.M./optical micrograph of the bonding interface between fibres and matrix: (**a**,**b**) SF and PF fibre in original FRC and after melting, (**c**) SF bridging effect, (**d**) microchannel due to melting of PF, (**e**,**f**) irregular void and microcracks in control.

SEM/optical analysis was done on the polish surface taken from the specimen’s core (up to 20 mm depth) to understanding the fibre–matrix interactions after repeated high temperatures and HC fluid exposures. Figure 16a shows PF fibres strewed in original FRC samples, and Figure 16b shows melted PF fibre due to high-temperature exposure. In the control specimen, repeated HC fluids exposure and high temperature create extra vapour pressure and heat-induced thermal cracks that may have caused the spalling (Figure 16e,f). Due to repeated exposure to 175 °C, there was a significant difference between PF and SF FRC porosity. When the PF fibre reinforced concrete was heated up to 175 °C, PF fibres start shortening in length due to relaxation, melting occurs. These micro-channels form a network of more permeable cement matrix, which contributes to the release of gas and water vapour, thus resulting in the reduction in the pore pressure [58,61]. This phenomenon helps to reduce the spalling tendency of concrete exposed to high temperature. Due to SF’s high melting temperature, the development of such a microchannel was not observed in SF reinforced concrete. However, SF’s presence helps bridge the cracks that develop due to repeated thermal exposures, as seen in Figure 16d.

For HB FRC, a better result was observed due to the combined effect of PF and SF, where melting of PF created a microchannel, which helps to release moisture pressure developed for HC fluids exposure. Simultaneously, SF’s presence helps prevent thermal cracks in concrete; thus, HB FRC showed better performance in high temperatures and HC fluid exposures.

## 4. Conclusions

Airfield concrete pavements face different severe loading conditions during their service life. The most significant one is repeated thermal shock from APU’s exhaust of jet engines and chemical attack from the leaked HC fluids. This effect causes the spalling of the concrete surface, which requires additional maintenance and replacement costs. Recent investigations showed that the inclusion of fibres improves the residual mechanical properties of concrete exposed to high temperatures and HC fluids. This study includes an experimental investigation on adding PF fibre and SF in the FRC mixes to improve the residual mechanical and thermal properties. The following conclusions are drawn as a result of this study.

- The compressive strength loss was more significant in specimens exposed to the combined high temperature and HC fluids than to the high temperature only. Specimens with PF showed slightly more strength loss than control specimens under similar loading conditions. The addition of SF resulted in more residual compressive strength than PF and control specimens. However, hybrid FRC performed much better in retaining residual compressive strength.
- The hybridisation of concrete using PF and SF was slightly more effective in enhancing the flexural properties of FRC than control and PF reinforced concrete. The specimens’ flexural strength decreased gradually with an increase in the number of high-temperature exposures due to the degradation of the tensile strength of fibres and bonding between the fibres and the matrix. Besides, PF’s melting created extra pores in the matrix to reduce the vapour pressure due to heating. Moreover, SF improves the FRC post-peak behaviour and reduced the crack propagation and the risk of spalling compared to OPC concrete.
- The thermal properties of FRC improved with the addition of SF and PF fibres. However, recurrent exposure to heat and HC fluids caused a gradual decrease in thermal conductivity and specific heat. Due to the excellent thermal conductivity, SF had the highest positive impact above HB FRC, and PF fibres exhibited the most negligible impact on thermal properties.
- Mass loss was prominent among PF and control samples than SF and HB samples. TG and DS test also confirmed the decomposition of concrete at the higher temperature besides loss of free and bound water, which has contributed to mass loss.
- Microstructure analysis revealed that the PF’s melting created extra pores in FRC that release the extra vapour pressure due to high-temperature exposures. The addition of SF reduces the microcracks propagation in the concrete by the bridging effect, thus enhancing the tensile capacity of FRC specimens.

In Brief, the above tests showed that the addition of fibres has a positive impact on the residual thermo-mechanical properties of FRC, except for PF fibre. Therefore, incorporating 0.7% HB fibres seems to be a promising way to enhance concrete resistance to thermally induced explosive spalling. In this experiment, concrete specimens were exposed to high temperatures from all directions, but the airfield APU exhaust applies one-directional heat. It will be worth considering that exposing concrete samples to one direction (1D) heating as it happens under the APU exhaust. It will reveal the effect of uneven thermal stress distribution developed from 1D exposure compared to this research’s all-directional heating.

## Figures and Tables

**Table 1:** Physical properties of general-purpose cement in Australia.

Physical Properties	Values	AS 3972 Limits
Fineness	402 m^2^/kg	-
Soundness	1 mm	5 mm (maximum)
Normal consistency	29.5%	-
Initial setting time	106 min	47 min (minimum)
Final setting time	181 min	Six hours (maximum)
Specific gravity	3.12	

**Table 2:** The properties of the OPC control sample at ambient temperature.

Properties of OPC	M70
Air Content (%)	4.15
Compressive strength (MPa)	78
Modulus of elasticity (GPa)	55
Indirect tensile strength (MPa)	5.5
Unit weight (kg/m^3^, hardened)	2452
Water Absorption capacity (%)	3.90

**Table 3:** Different properties of PF fibre and SF (as per manufacturer specification).

Sample	Shape of Fibre	Length *l* (mm)	Diameter *d* (mm)	Aspect Ratio *l*/*d*	Density (kg/m^3^)	Tensile Strength (MPa)	Elastic Modulus GPa
PVA	Straight	12	0.038	210	1300	1620	42.5
MSF	Straight	8	0.22	35	7800	2000	200

**Table 4:** Mix design of concrete mixes.

Mix No.	Mixture ID	W/B	Water	Cement	Fine Agg.	Coarse Agg.	* Super Plasticiser	Fibre Volume Fraction (%)	
			(kg/m^3^)					PF.	**SF**
1.	Control	0.42	220	540	688	1030	-	-	-
2.	0.3%SF	0.42	220	540	688	1030	5.0	-	0.3
3.	0.3% PF	0.42	220	540	688	1030	6.0	0.3	-
4.	0.3% HB	0.42	220	540	688	1030	6.5	0.3	0.3
5.	0.5% SF	0.42	220	540	688	1030	5.2	-	0.5
6.	0.5% PF	0.42	220	540	688	1030	6.5	0.5	-
7.	0.5% HB	0.42	220	540	688	1030	6.5	0.5	0.5
8.	0.7% SF	0.42	220	540	688	1030	5.2	-	0.7
9.	0.7% PF	0.42	220	540	688	1030	7	0.7	-
10.	0.7% HB	0.42	220	540	688	1030	7	0.7	0.7

* Percentage of the total weight of cementitious material.

**Table 5:** Mass loss in the different concrete specimens after heat and HC exposures.

Temperature (°C)	Released Content	Types of Fibre	PF EO	SF EO	HB EO	PF E6O	SF E6O	HB E6O
20–200	H_2_O	Control	3.19	3.19	3.19	2.92	2.92	2.92
		0.3%	3.34	2.74	3.80	1.15	1.40	0.90
		0.5%	2.81	3.41	4.22	1.34	1.32	1.10
		0.7%	3.93	-	3.33	1.49	-	1.37
200–350	C-S-H	Control	0.82	0.82	0.82	2.5	2.50	2.50
		0.3%	1.27	0.82	1.49	2.69	2.73	2.15
		0.5%	1.12	1.26	1.72	3.23	2.77	2.73
		0.7%	1.59	-	1.43	4.33	-	2.74
350–500	OH	Control	1.13	1.13	1.13	3.17	3.17	3.17
		0.3%	0.99	0.98	1.21	2.95	2.90	3.67
		0.5%	1.00	1.34	1.44	5.11	3.52	3.18
		0.7%	1.48	-	1.28	5.54	-	3.49
500–800	CO_2_	Control	2.03	2.03	2.03	3.64	3.64	3.64
		0.3%	4.77	3.00	5.13	3.88	3.74	4.11
		0.5%	2.87	3.76	4.88	4.46	3.49	3.65
		0.7%	3.13	-	3.48	4.58	-	4.26

## Data Availability

The data presented in this study are available on request from the corresponding author.
